# Cystoid edema, neovascularization and inflammatory processes in the murine Norrin-deficient retina

**DOI:** 10.1038/s41598-018-24476-y

**Published:** 2018-04-13

**Authors:** Susanne C. Beck, Marcus Karlstetter, Marina Garcia Garrido, Yuxi Feng, Katharina Dannhausen, Regine Mühlfriedel, Vithiyanjali Sothilingam, Britta Seebauer, Wolfgang Berger, Hans-Peter Hammes, Mathias W. Seeliger, Thomas Langmann

**Affiliations:** 1 Division of Ocular Neurodegeneration, Institute for Ophthalmic Research, Centre for Ophthalmology, Tuebingen, Germany; 2 Laboratory for Experimental Immunology of the Eye, Department of Ophthalmology, University of Cologne, D-50931 Cologne, Germany; 3 Bayer AG, Wuppertal, Germany; 4 Institute of Experimental and Clinical Pharmacology and Toxicology, Medical Faculty Mannheim, University of Heidelberg, D-68169 Mannheim, Germany; 5 Institute of Medical Molecular Genetics, University of Zurich, Zurich, Switzerland; 6 Center for Integrative Human Physiology (ZIHP), University of Zurich, Zurich, Switzerland; 7 Neuroscience Center Zurich (ZNZ), University and ETH Zurich, Zurich, Switzerland; 8 5th Medical Department, Medical Faculty Mannheim, University of Heidelberg, D-68169 Mannheim, Germany

## Abstract

Mutations in the Norrin (NDP) gene cause severe developmental blood vessel defects in the retina leading to congenital blindness. In the retina of Ndph-knockout mice only the superficial capillary network develops. Here, a detailed characterization of this mouse model at late stages of the disease using *in vivo* retinal imaging revealed cystoid structures that closely resemble the ovoid cysts in the inner nuclear layer of the human retina with cystoid macular edema (CME). In human CME an involvement of Müller glia cells is hypothesized. In Ndph-knockout retinae we could demonstrate that activated Müller cells were located around and within these cystoid spaces. In addition, we observed extensive activation of retinal microglia and development of neovascularization. Furthermore, *ex vivo* analyses detected extravasation of monocytic cells suggesting a breakdown of the blood retina barrier. Thus, we could demonstrate that also in the developmental retinal vascular pathology present in the Ndph-knockout mouse inflammatory processes are active and may contribute to further retinal degeneration. This observation delivers a new perspective for curative treatments of retinal vasculopathies. Modulation of inflammatory responses might reduce the symptoms and improve visual acuity in these diseases.

## Introduction

Norrin signalling is essential for correct vascular development. Mutations in the *NDP* (Norrie disease pseudoglioma) gene that is encoding the Norrie protein^1^ cause Norrie disease which is characterized by progressive deafness, mental retardation and congenital blindness^2^. The respective mouse model, generated in 1996 by homologous recombination in embryonic stem cells (Ndph^y/−^ mouse)^3^, develops symptoms similar to those observed in Norrie disease patients^4–6^. During development, the defects in sprouting angiogenesis lead to abnormal vessel growth with a complete lack of deeper retinal capillaries along with a delayed hyaloid vessel regression that result in persistent vitreoretinal membranes^7–9^. Recently, by investigating the long-term consequences of Norrin deficiency we observed a close interplay between the primary developmental defects and secondary alterations that affected the constituents of the retinal vasculature and resulted in the development of microaneurysm-like lesions with extensive vascular fenestration^9^.

In the present work, we observed severe alterations in the retinal layer morphology. Surprisingly, we detected a large number of cystoid lesions spread across the entire retina that closely resemble the retinal alterations of patients suffering from cystoid macular edema (CME)^10,11^. CME occurs in various human retinal diseases like AMD, diabetic retinopathy, retinal vein occlusion, retinitis pigmentosa^10,12^, optic atrophy^13^ or open-angle glaucoma^14^. Characteristic of CME are cyst-like spaces that accumulate radially within the perifoveal region^11^ mainly in the inner nuclear layer (INL) of the retina^10^. The presence of cysts causes a thickening of the perifoveal retina and decreases the visual acuity^11,15^. Moreover, the compression of the neuroretina, the nerve fibers and capillaries by the cystic alterations further contributes to retinal degeneration and aggravation of hypoxic conditions.

Different factors like osmotic and hydrostatic forces, together with capillary permeability and tissue compliance ensure the fluid homeostasis of the retina. Any insult to this fine-tuned balance leads to cystic alterations that can be caused by either extra- or intracellular edema or both^10^. In the macula the high neuronal activity demands a high metabolic rate which both are accompanied by strong water accumulation. Also the intraocular pressure continuously forces water into the retina. Therefore, to ensure homeostasis, a constant water efflux out of the retinal tissue into the blood is necessary. In the healthy retina Müller glia cells continuously dehydrate the inner retina^16^. The water transport is accomplished by a complex interplay of aquaporin water channels and Kir4.1 potassium ion channels^17^. Pathological conditions, however, alter the expression pattern of water channels leading to impaired Müller cell function and finally to edema development^16^. Thus, Müller glia cells are believed to play a key role in the pathophysiology of CME^17^. In the present work we were therefore particularly interested in the function of Müller cells in the ageing Norrin deficient retina. Concretely, very similar to the pathophysiology of CME, we could directly associate activated Müller cells with the observed intraretinal cystic lesions. Moreover, we observed neovascularization and widespread inflammatory processes involving Müller cell gliosis and microglia activation. These observations give a new impact to the mouse model of Norrie disease. On the basis of our results we hypothesize that inflammation might be a common process that occurs in the chronic phase in many ocular diseases irrespective of the initial pathological process.

## Results

### Vascular alterations in the ageing Norrin deficient retina

**Figure 1:**
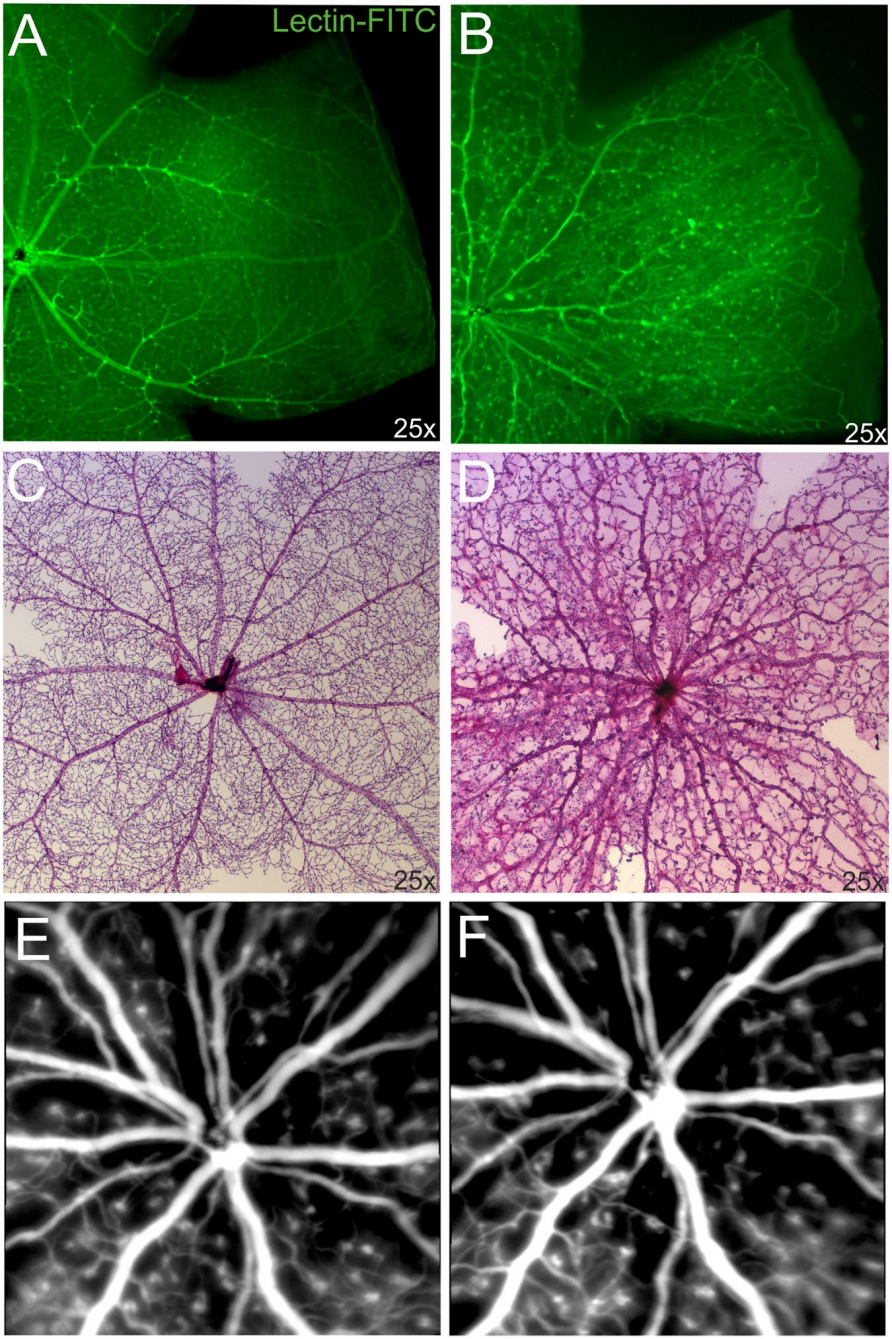
Vascular alterations of the superficial plexus in adult Ndph^y/−^ mice. Lectin-FITC-stained retinal whole mount preparations of 2 months old control (**A**) and age matched Norrin deficient mice (**B**) detected reduced outgrowth of the retinal primary plexus. (**C**,**D**) Analysis of the retinal vasculature and capillary network by PAS stained retinal digest preparations revealed severe vascular alterations in the retina of 2 months old Ndph^y/−^ mice (**D**) compared to age matched control (**C**). (**E**,**F**) Long-term monitoring by *in vivo* SLO imaging (ICG angiography) between 2 months (**E**) and 5 months of age (**F**) did not reveal further overall vascular alterations. SLO images of the same individual are shown.

**Figure 2:**
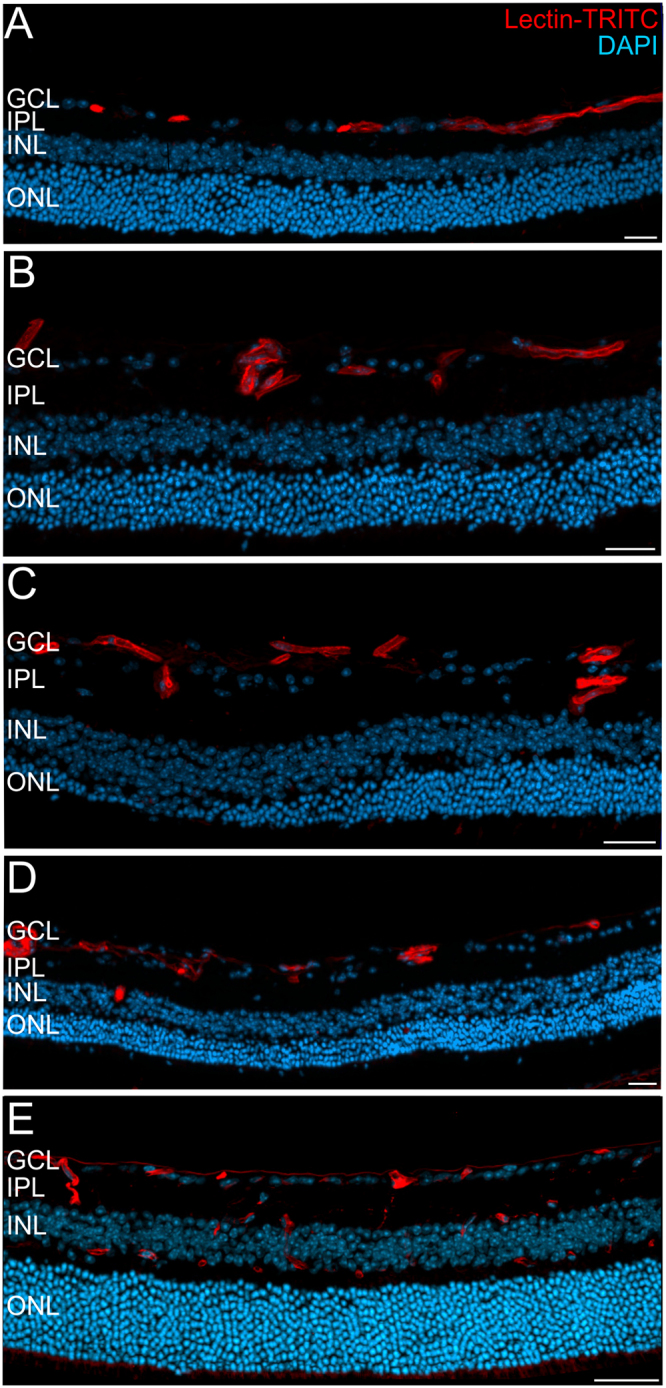
Defects in the retinal vasculature in the Ndph^y/−^ retina at 18 months of age. Cryosections were labelled with Lectin-TRITC (red) for detection of vascular structures and counterstained with DAPI (blue). (**A**–**D**) In representative sections of two Ndph^y/−^ mice the superficial vasculature at the level of the GCL and large microangiopathies extending into the INL (**B**,**C**) were detected. Although a single vessel could be observed at the level of the INL (**D**) the Norrin deficient retina remained virtually avascular until 18 months of age. (**E**) In age matched WT animals all three vascular layers that extend deep into the neuroretina were present. GCL: ganglion cell layer, IPL: inner plexiform layer, INL: inner nuclear layer, ONL: outer nuclear layer. Scale bar, 50 µm.

Under normal developmental conditions the retina of adult mice is completely vascularized and three layers of capillaries are present (Fig. 1A,C). In the adult Norrin deficient retina the primary superficial capillary network had completely transformed into drum-stick like microangiopathies (Fig. 1B,D) and the retina was still not completely vascularized (Fig. 1B). As shown previously, at two months of age the vessels of the Norrin deficient retina were still confined to the ganglion cell layer (GCL) and inner plexiform layer (IPL), deeper retinal capillaries were not present leaving the retinal layers completely avascular and thus resulting in tissue hypoxia^9^. In our previous work we also observed vascular remodelling until 2 months of age^9^. To analyse if these modifications further progressed, we made use of *in vivo* SLO imaging that allows for the long-term monitoring of the same individual^18^. We repeatedly examined Norrin deficient mice until 5 months of age. Between 2 and 5 months no further vascular remodelling could be observed in the overall appearance of the superficial vascular plexus (Fig. 1E,F). Additionally, histological analyses were performed at 18 months of age (Fig. 2). Lectin staining revealed that the majority of the vessels were still confined to the GCL (Fig. 2A). The microangiopathies could still be detected and appeared as densely accumulated vascular structures that extended deep into the IPL (Fig. 2B,C), and, very occasionally, even into the INL (Fig. 2D). This observation indicated to ongoing vascular remodelling, at least at isolated sites. Since these vascular protrusions were restricted to only single sites the Norrin deficient retina could still be considered as avascular at 18 months of age.

### Activation of glial cells in the retina of adult Ndph^y/−^ mice

**Figure 3:**
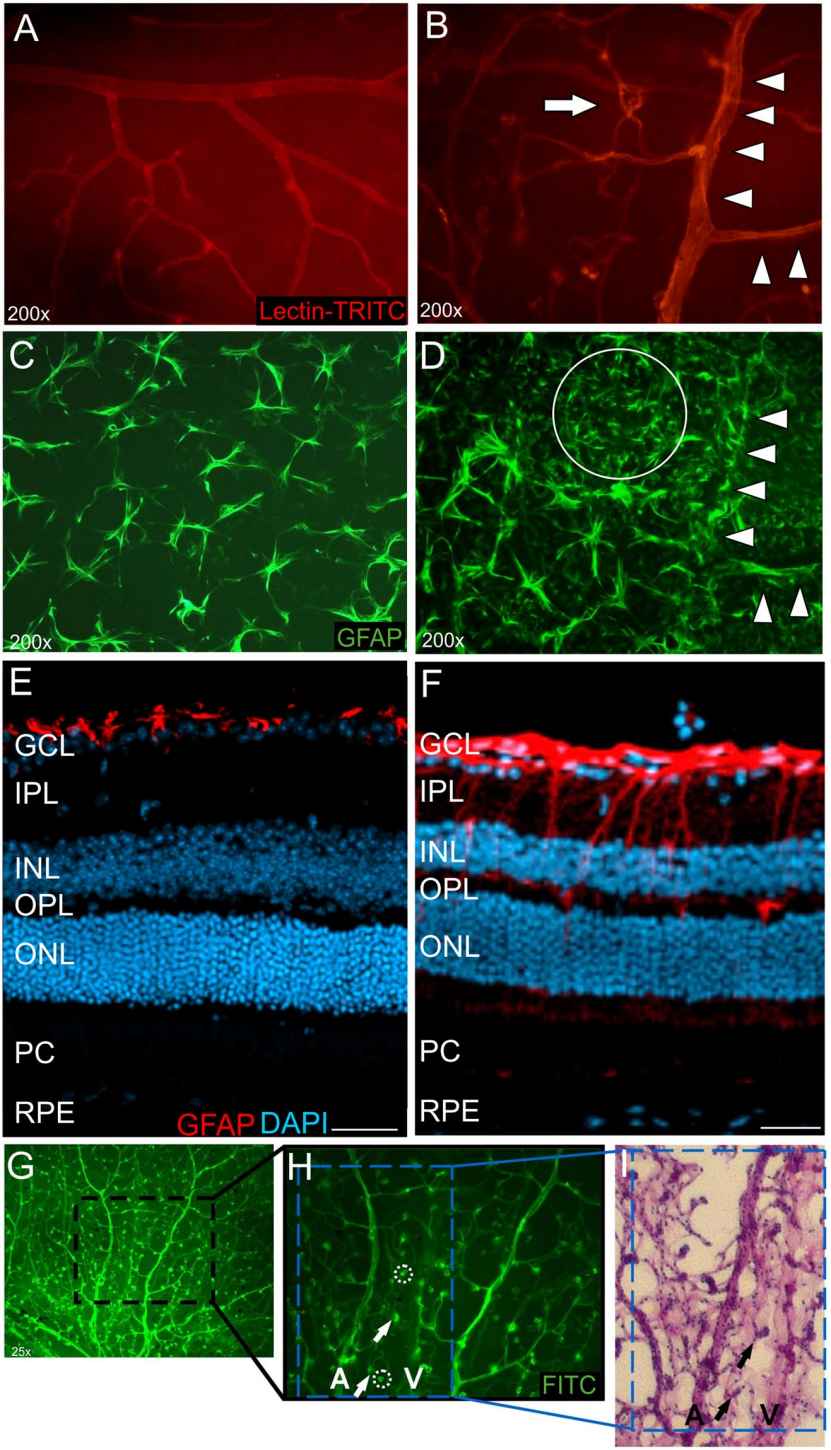
Activation of glial cells in the adult Ndph^y/−^ retina at two months of age. (**A**–**D**) retinal whole mount preparations labelled for the detection of retinal vasculature with isolectin in WT (**A**) and mutant (**B**) animals. GFAP staining revealed large quantities of astrocytes covering the retina of Ndph^y/−^ mice (**D**) compared to control (**C**). (**E**,**F**) retinal cryosections stained for GFAP revealed extensive gliosis and activated Müller cells that span the entire retina of mutant animals (**F**) whereas in WT animals gliosis were not observed (**E**). GCL: ganglion cell layer, IPL: inner plexiform layer, INL: inner nuclear layer, OPL: outer plexiform layer, ONL: outer nuclear layer, PC: photoreceptors, RPE: retinal pigment epithelium. Scale bar, 50 µm. Cells of the mononuclear phagocyte system accumulate in the extravascular space in the retina of 2-months-old Ndph^y/−^ mice. (**G**) FITC-stained retinal whole mount overview, (**H**) magnification. (**I**) retinal digest preparation. Some cells that were present on the FITC-stained retinal whole mounts (arrows and rings) disappeared after retinal digestion (rings) whereas others were still recognizable (arrows), thus indicating that cells of the mononuclear phagocyte system moved outside the vascular compartment to be secondarily eliminated by the retinal digestion step. A: artery, V: vein.

In healthy conditions glial cells support the retinal integrity and constitute a functional link between neurons and vessels. Retinal capillaries are closely covered by glial cell processes arising from astrocytes and Müller cells^17^. Since pathogenic stimuli activate glial cells^19^, we analysed whether the extensive vascular alterations and persistent tissue hypoxia also affected the glial cells in the retina of Ndph^y/−^ mice (Fig. 3). Isolectin/GFAP double stainings on whole mounted wild-type (Fig. 3A,C) and mutant retinas (Fig. 3B,D) revealed that cells with the typical stellar morphology of astrocytes were present in large number in Ndph^y/−^ mice since they covered the entire retinal surface (Fig. 3D). In the control animals the astrocytes were evenly distributed (Fig. 3C), whereas in the Norrin deficient retina the astrocytes (Fig. 3D, circle) were particularly clustered in areas with microangiopathies (Fig. 3B, arrow) and predominantly oriented along the large retinal vessels (Fig. 3B,D; arrow heads). Moreover, GFAP-stained retinal cryosections of Ndph^y/−^ mice revealed extensive reactive gliosis of Müller cells spanning the entire retina (Fig. 3F), whereas under normal conditions activated Müller cells were not observed (Fig. 3E).

Lectins label retinal vasculature and cells of the mononuclear phagocyte system of the CNS^20,21^. While studying the outgrowth of retinal superficial capillaries on Lectin-FITC-stained retinal whole mounts (Fig. 1A,B) on one hand and analysis of retinal vessels on digested retinal whole mounts stained with PAS on the other hand (Fig. 1C,D), we observed an accumulation of cells of the mononuclear phagocyte system in the retina of Ndph^y/−^ mice (Fig. 3G–I). These cells were present in large numbers in the extravascular space exclusively in Ndph^y/−^ retinas, whereas in wild type control retinas these cells could not be detected. In comparison to the Lectin-FITC-stained retinal wholemounts (Fig. 3G,H) some of these particular cells were absent on the retinal digest preparations (Fig. 3I). This observation led to the suggestion that these cells may have extravasated the vascular compartment. Due to the perivascular location these cells were then secondarily eliminated by the retinal digestion step. Such an accumulation was not observed in the retina of control animals. Attraction to particular locations and extravasation of leukocytes, like cells of the monocyte-macrophage family, are typical signs of tissue inflammation and damage further indicating to pathologic processes ongoing within the Ndph^y/−^ retina^22^. Under pathological conditions like CME, the migration of leucocytes to the retinal tissue were directly be correlated to the breakdown of the blood retina barrier (BRB)^10,23^.

### *In vivo* analysis of vascular microangiopathies and retinal layer morphology

**Figure 4:**
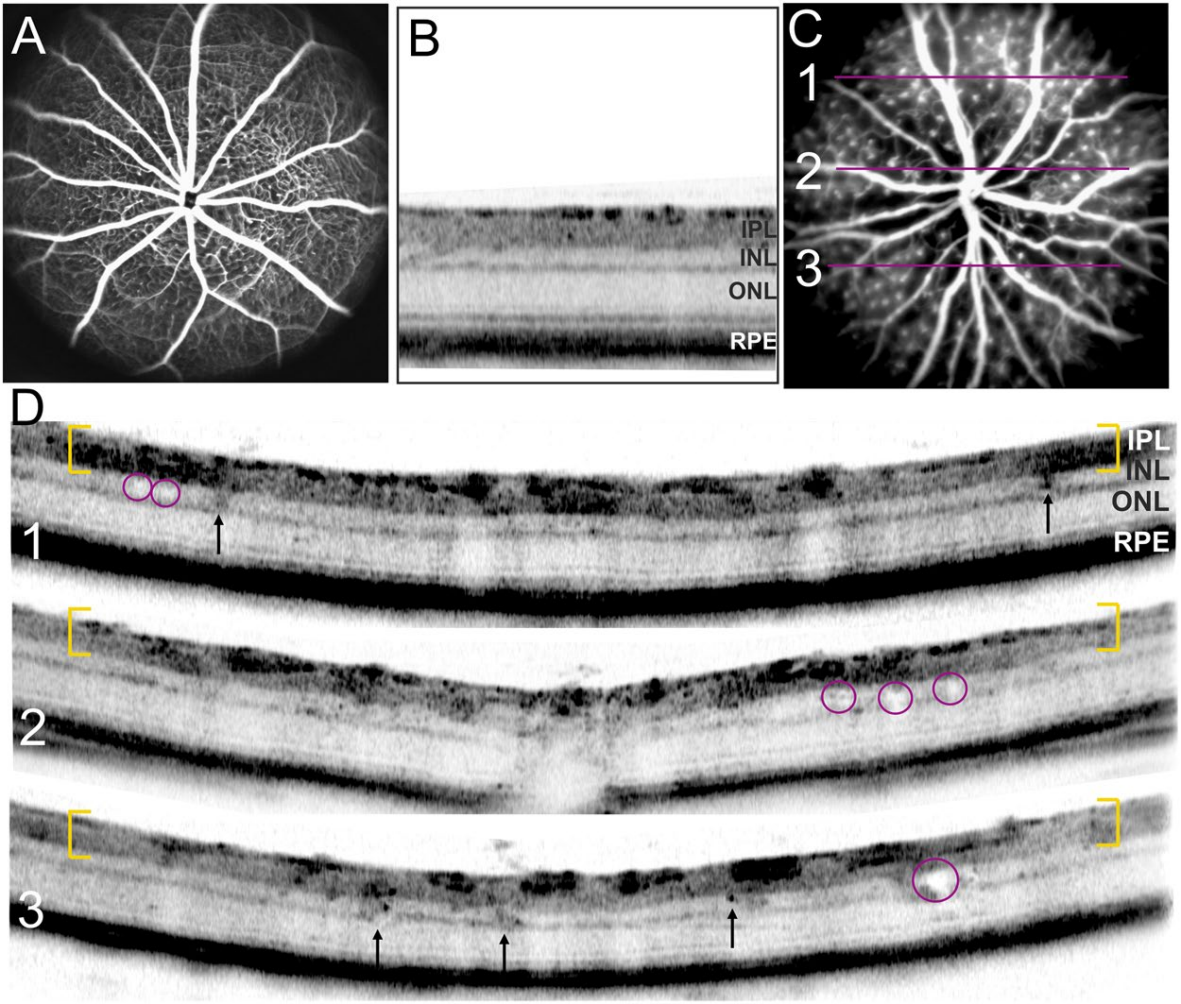
*In vivo* analysis of microangiopathies and retinal layer morphology at two months of age. SLO imaging of WT (**A**) and mutant animals (**C**) with *in vivo* angiography (ICG). OCT imaging of WT mice displayed the regular structure of retinal layering (**B**) whereas in mutant mice severe changes of retinal layer morphology could be observed (**D**). Yellow brackets depict the GCL and IPL covered with microaneurysm-like lesions, red circles highlight retinal microcysts, focal hyperreflective alterations within the INL are indicated by arrows. The fundus picture (**C**) indicates the orientation of the respective cross-sectional OCT scans (**D**).

*In vivo* angiography allows the detection of the large retinal vessels as well as the capillaries as shown in normal WT animals (Fig. 4A). In adult Ndph^y/−^ mice, however, the entire vasculature was covered by microangiopathies (Fig. 4C) which have already been analysed in detail recently^9^. Examination of retinal layer morphology with *in vivo* imaging revealed that these microaneurysm-like angiopathies were also detectable by OCT analyses (Fig. 4D). As described previously^24^, in OCT sections of WT animals vessels present as dark, highly reflective roundish structures in the inner part of the retina (Fig. 4B). In mutant animals however, the microangiopathies were clearly distinguishable as large knob-like structures in the ganglion cell layer and inner plexiform layer (Fig. 4D, yellow brackets) and they were present across the entire retina visualized by OCT. Surprisingly, OCT analyses detected not only the vascular alterations but revealed also severe changes in retinal layer morphology. In particular, many cyst-like lesions were discovered within the inner nuclear layer (Fig. 4D, red circles).

Moreover, a lot of dark-appearing (hyperreflective) focal alterations within the INL were scattered across the entire fundus (Fig. 4D, arrows). Since Norrin dependent defects are characterized by the complete lack of deeper retinal capillaries, still at 2 months of age^9^, these hyperreflective alterations can not correspond to vascular structures. Noticeably, these alterations were also evenly distributed throughout the entire retina.

### The intraretinal microcysts were correlated to activated Müller cells

**Figure 5:**
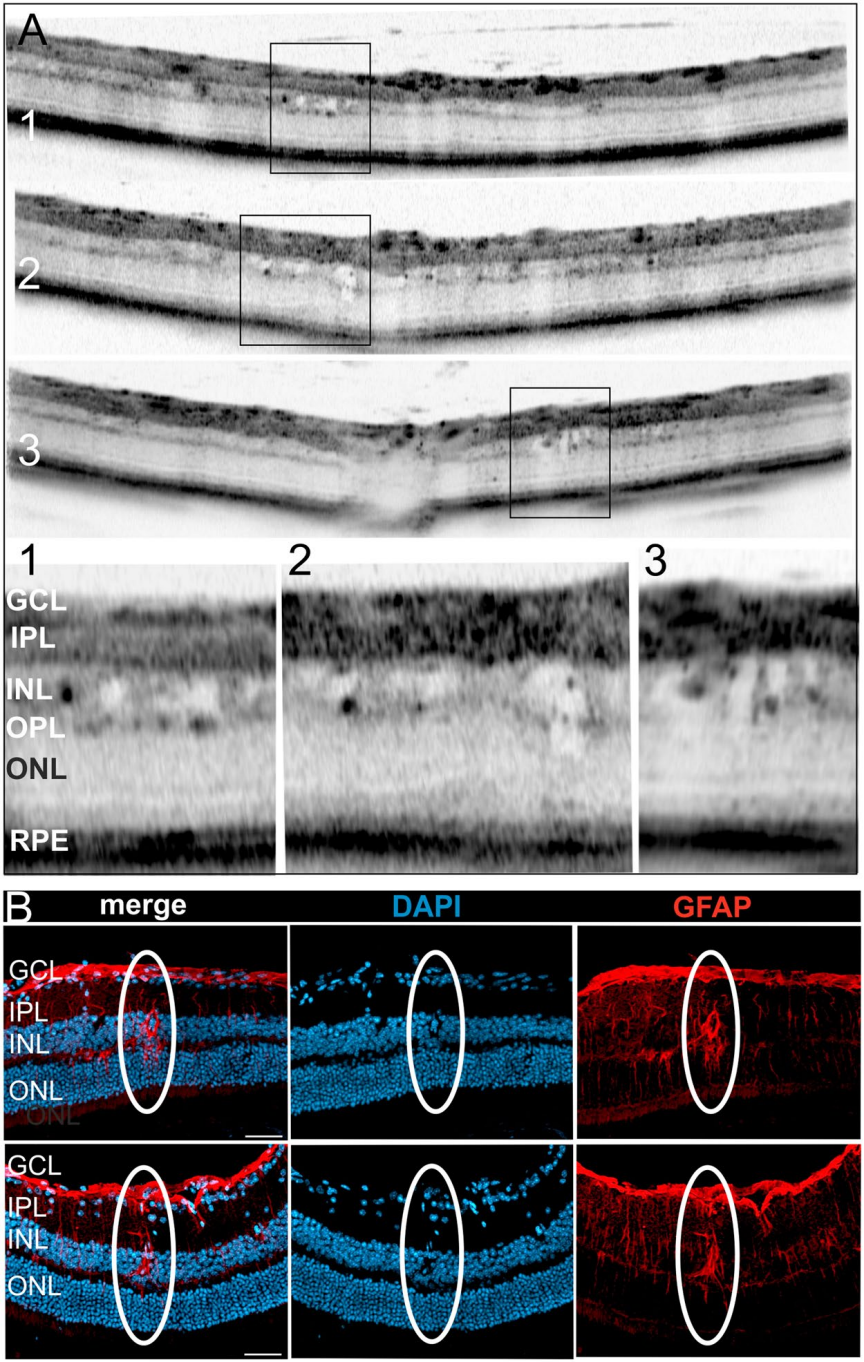
Detailed analysis of intraretinal microcysts. (**A**) Representative OCT sections show several intraretinal cysts in two months old mutant animals. The regions of interest are depicted by rectangles with the respective magnifications numbered correspondingly. (**B**) Histological sections stained with DAPI and GFAP to detect activated Müller glia cells. Overlay of the labelling (merge) clearly revealed the direct correlation of the Müller cell with the intraretinal cyst, two representative cysts are shown. Scale bar, 50 µm.

**Figure 6:**
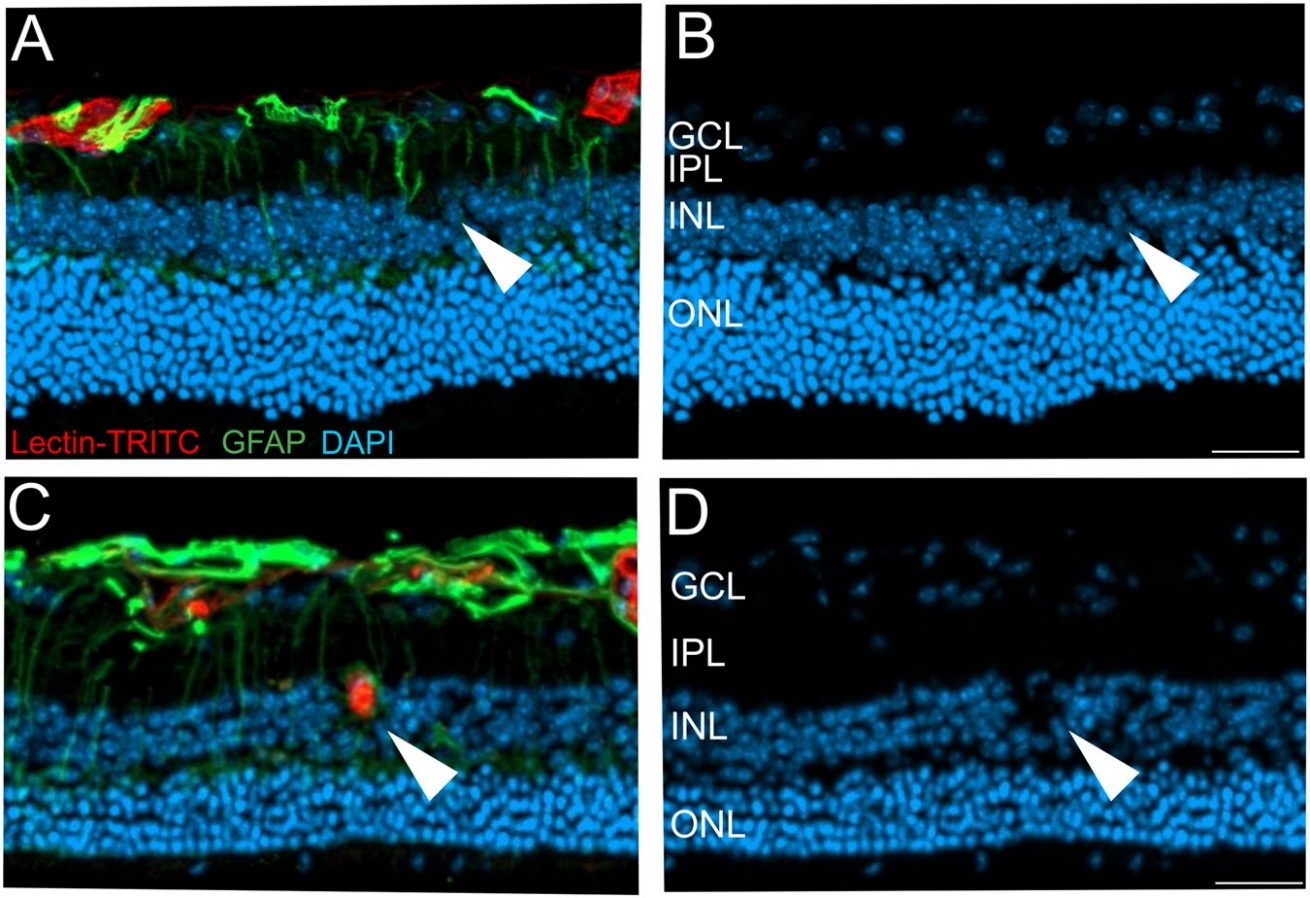
Intraretinal microcysts in relation to the Norrin dependent vascular defects. (**A**,**C**) Histological sections of the Norrin deficient retina at 18 months were labelled with Lectin-TRITC (red) and GFAP (green) to detect vascular structures and activated Müller cells, respectively. DAPI staining (blue) revealed retinal cystic alterations (**B**,**D**). (**A**,**C**) As also shown in Fig. 2 the Norrin dependent vascular alterations leave the retina virtually avascular, thus the microcystic alterations were almost exclusively correlated to activated Müller cells (**A**,**B**, arrow heads and Fig. 5). However, a single vessel extending into the INL (**C**) was found to be correlated to a cystic lesion (**D**) and an activated Müller cell (**C**). Scale bar, 50 µm.

Next, we were interested in a more detailed analysis of the observed lesions within the INL. High resolution OCT imaging and magnification of the regions of interest (Fig. 5A, OCT sections and corresponding magnifications 1–3) clearly depicted several fluid-filled microcysts. Very often, fluid accumulation within the tissue does not endure histological procedures, however, when present, OCT reliably allows the detection of edema^25^. In OCT imaging the dimension as well as the shape of the observed intraretinal cysts very much resemble the cystic alterations that were present in patients suffering from cystoid macular edema (CME)^10,12–14^. Since so many cystoid structures were present in the Norrin deficient retina (Fig. 5A) we were able to detect lesions also in histological sections (Fig. 5B). DAPI labelling revealed the displacements of cells leaving a cyst-like space within the INL of the mutant retina. Representative lesions are shown in Fig. 5B. Moreover, these cysts could directly be correlated to activated Müller cells (Fig. 5B and Fig. 6A,B).

As reported previously in 2 months old animals^9^ and as additionally demonstrated in very aged animals at 18 months (Fig. 2) the vasculature of the Norrin deficient retina was restricted almost entirely to the GCL and IPL leaving the retina virtually avascular. However, at 18 months of age a single vascular extension into the INL was observed. Performing lectin/GFAP double staining this vessel could be correlated to a cystic lesion and an activated Müller cell (Fig. 6C,D). Here, it would also be possible that the malfunction of the vascular structure caused local edema that displaced the cells and finally created the cystic space observed. These findings very much resemble the pathophysiology of CME. Here, the cystic fluid accumulation is correlated to either edema caused by vascular dysfunction or the impaired function of Müller cells or both^10,16,17^.

### Activation and proliferation of retinal microglial cells

**Figure 7:**
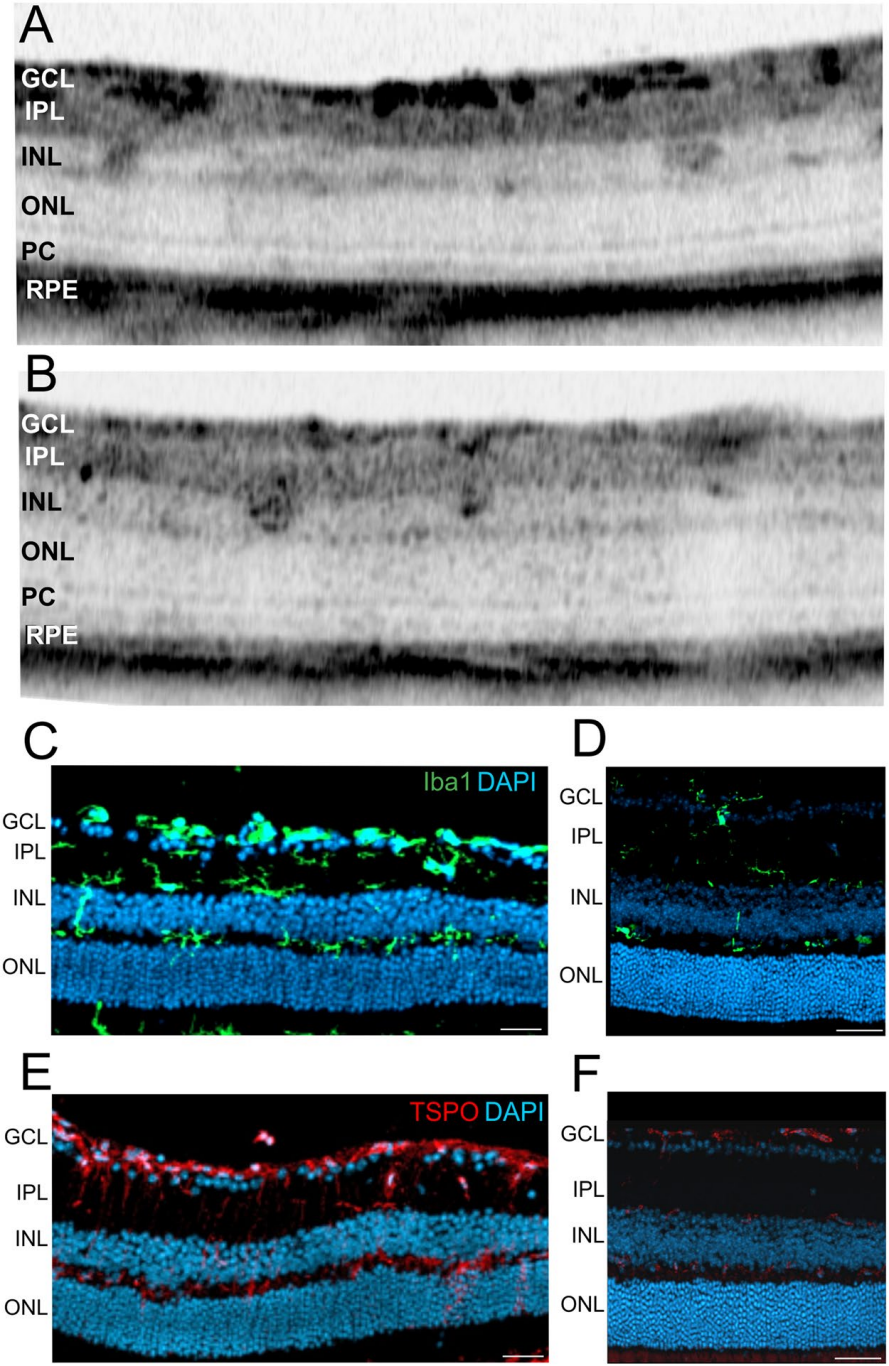
Focal morphology alterations detected in the retina of two months old mutant mice by OCT imaging and immunostaining of histological sections for activated microglial cells. (**A**,**B**) Magnifications of representative OCT sections show several focal alterations within INL and IPL. (**C**–**F**) Immunohistological sections stained for Iba1 (**C**,**D**, green) of Ndph^y/−^ mice (**C**) and age matched control animals (**D**), stained for TSPO (E, F, red) of Ndph^y/−^ mice (**E**) and control animals (**F**). DAPI, blue. Scale bar, 50 µm.

In a next step we analysed the dark focal alterations (Fig. 4D, arrows) within the INL that were also very abundant and could also be found throughout the entire retina. Since the INL of the adult Norrin deficient retina was shown to be devoid of capillaries^9^ (Fig. 2), vascular structures could be excluded. In magnifications of OCT sections (Fig. 7A,B) irregular morphological alterations could be observed and those were not always confined to the INL but also extended into the IPL and/or OPL.

Since GFAP staining revealed excessive retinal gliosis concerning Müller glial cells (Fig. 3F) and astrocytes (Fig. 3D), we were interested in the microglial cells within the mutant Norrin deficient retina. Immunohistological analyses revealed extensive proliferation of microglial cells throughout the entire retina (Fig. 7C) compared to control (Fig. 7D). Moreover, TSPO-staining revealed high activation of microglial cells (Fig. 7E). Under normal conditions, (Fig. 7D,F) microglial cells can only be found in the IPL and OPL, in the Norrin deficient retina microglial cells were detected also in the INL and ONL (Fig. 7C,E). Moreover, during the homeostatic phase of the retina, microglial cells present a stellar, highly ramified shape, during activation and in the effector state, microglial cells transform to large amoeboid like structures^26,27^.

In the past decade OCT *in vivo* imaging has evolved as a fundamental technology that not only complements *ex vivo* histomorphology but give new information about retinal structures and additional insights into retinal layer morphology^24,25,28–30^. In this field, several studies have proven OCT scans to be highly consistent with histological sections in similar resolution^24,25,31,32^, therefore it is common standard in ophthalmic research to draw conclusions from the comparison of OCT imaging to data from histological sections. Thus, on the basis of the specific pathology of the Norrin deficient retina and according to location, number, size and shape of the focal alterations observed in OCT sections we hypothesize that these structures are activated microglial cells visible by *in vivo* OCT imaging (Fig. 7A,B).

### Hot spots of infiltrating activated microglial cells were present in the outer retina

**Figure 8:**
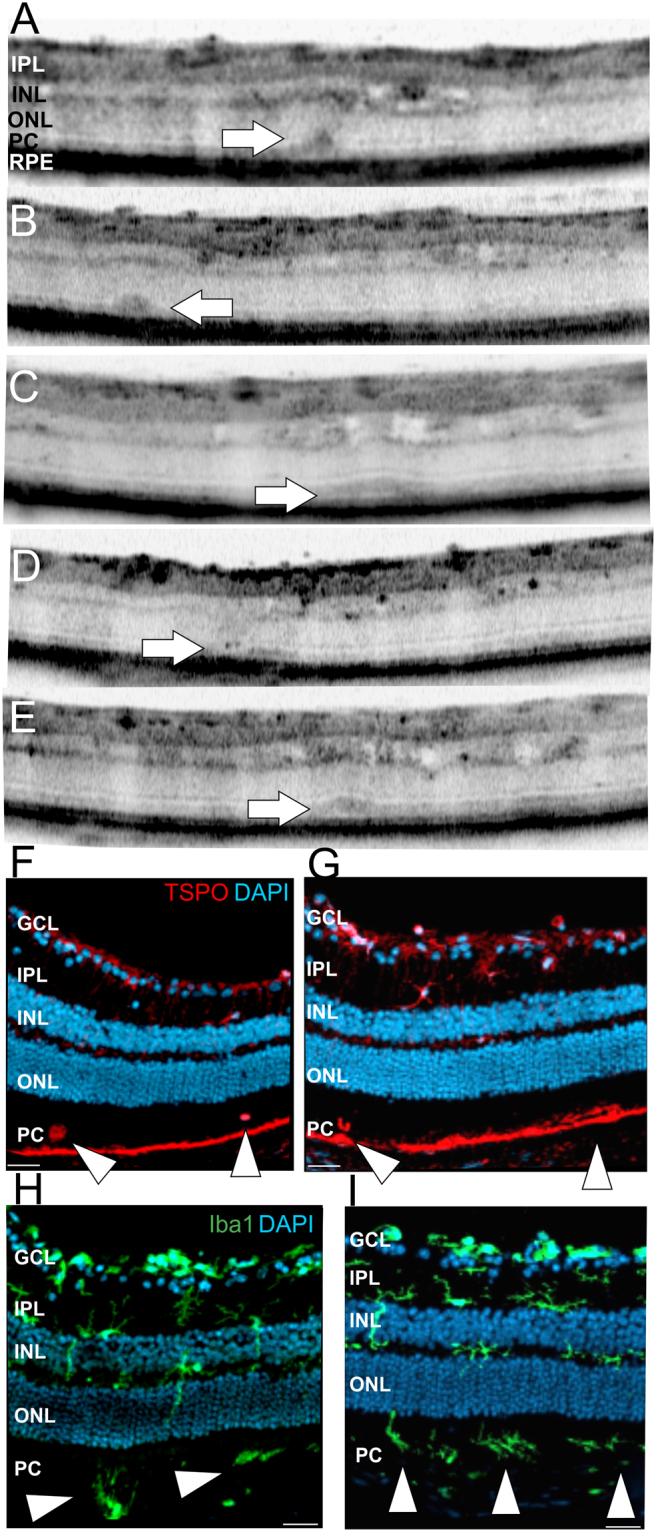
Detection of subretinal defects by OCT imaging and analysis of activated microglial cells in immunohistological sections in two months old Ndph^y/−^ mice. (**A**–**E**) Magnifications of representative OCT sections show several focal layer morphology alterations within the photoreceptor layer (arrows). (**F**–**I**) Representative immunohistological sections stained for TSPO (**F,G**, red) and Iba1 (**H,I**, green). DAPI, blue. Scale bar, 50 µm.

Further analyses with OCT imaging revealed changes of retinal layer morphology also in the outer retina. In magnifications of OCT sections several sites of focal displacement of photoreceptor cells could be detected (Fig. 8A–E, arrows). In Fig. 8A it appears that the large focal alteration within the photoreceptor cell layer extends into the inner retina. This observation directly led to the suggestion that microglial cells might have migrated into the outer retina. Indeed, histological sections (Fig. 8F–I, arrow heads) corroborated this result. Microglial cells could be detected within the photoreceptor layer both with Iba1 staining (Fig. 8H,I, green) and TSPO staining (Fig. 8F,G) for activated microglial cells. Moreover, from *in vivo* OCT imaging it could be suggested that the infiltrating microglial cells have even induced local retinal detachment (Fig. 8C,E, arrows). Thus, we suggested that the different appearances of these lesions represent different stages of microglia cells that have infiltrated the outer retina and now interfere with layer morphology and layer integrity.

### Formation of outer retinal neovascularization

**Figure 9:**
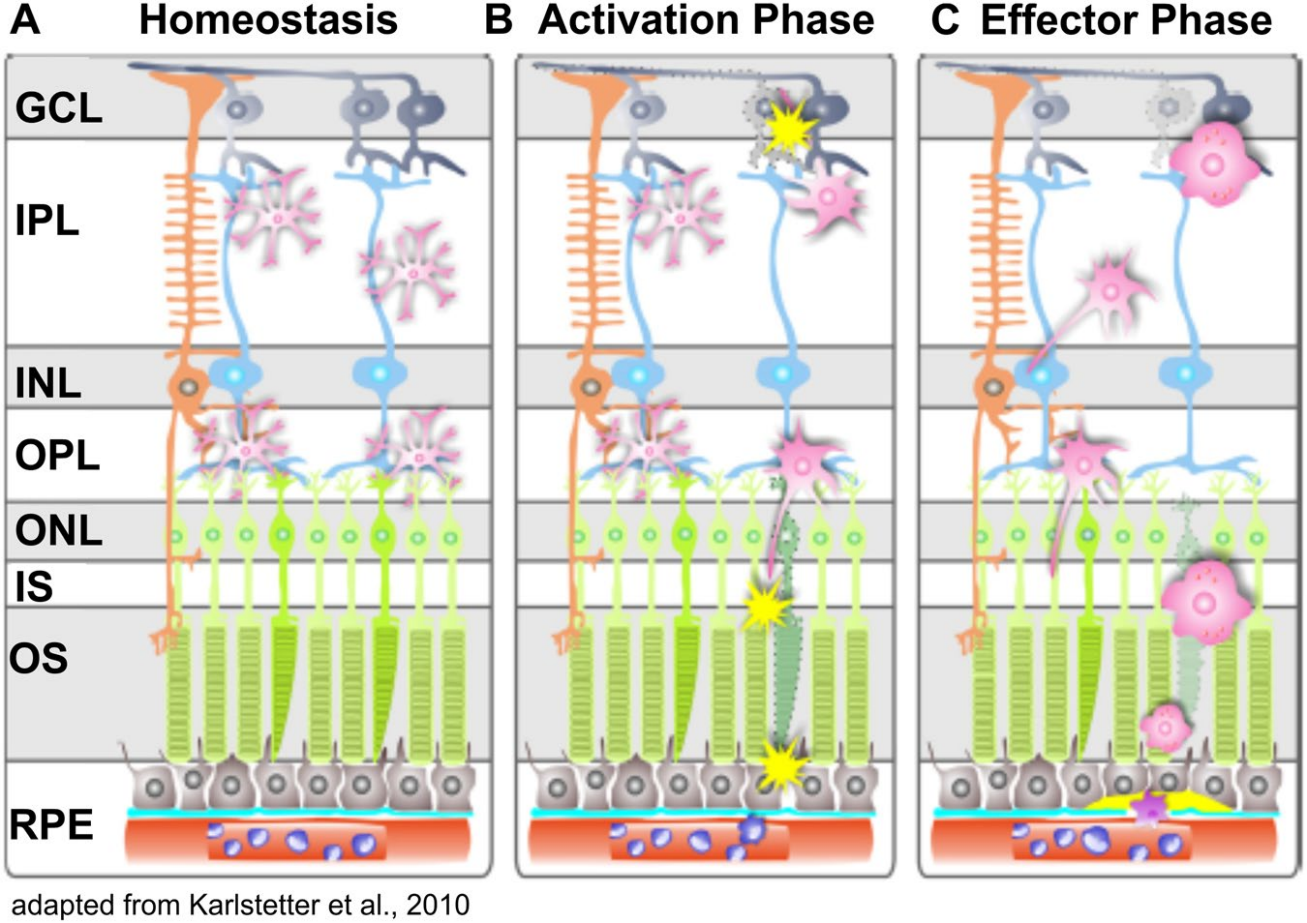
Schematic overview of microglial activity in the retina. (**A**) Under normal conditions, ramified microglia are mainly present in the plexiform layers. They continuously surveil the retinal layers with their long protrusions. (**B**) Abnormal cell functions or degeneration in the retinal layers rapidly activate microglia and they transform into large amoeboid effector cells (**C**). Modified from Karlstetter *et al*.^26^.

Although no further alterations of the overall retinal vasculature between 2 and 5 months of age were detected (Fig. 1E,F), the persistent tissue hypoxia had obviously triggered inflammatory processes that resulted in severe alterations of retinal layer morphology also in the outer retina. Here, these effects could be associated to activated microglial cells (Fig. 8). The different appearances of the infiltrating cells could be suggested to reflect various stages of microglial activation (Fig. 9).

**Figure 10:**
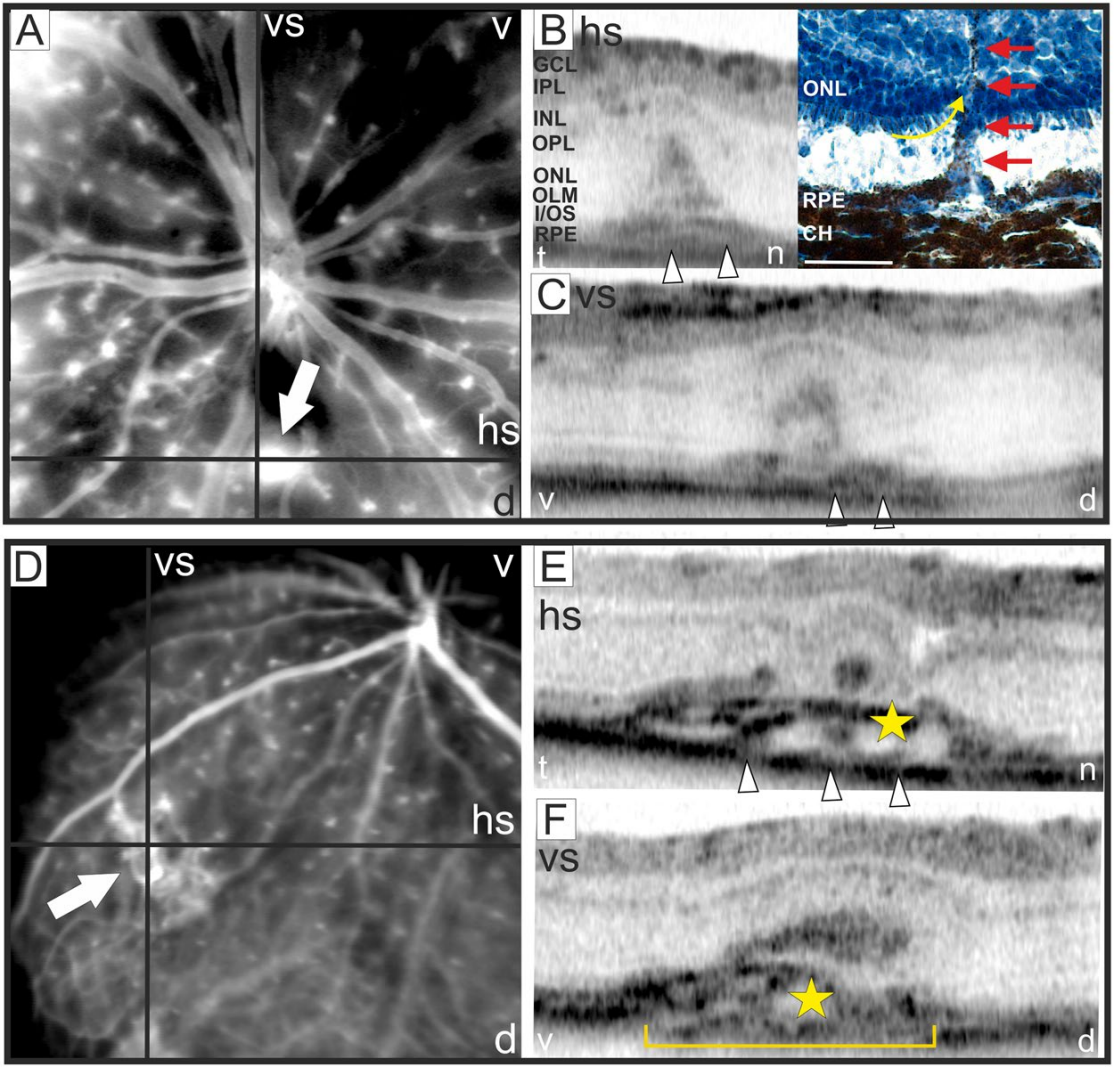
Detection of neovascularization. At 3 months of age in two out of five eyes NV could be observed. (**A**,**D**) SLO angiography with FL, the position of the respective OCT scans (**B**–**F**) are depicted. V: ventral, d: dorsal, t: temporal, n: nasal, hs: horizontal scan, vs; vertical scan. In (**B**) OCT scan (left) with the corresponding retinal cryosection stained with hematoxylin (right) is shown. The histomorphology at 18 months of age corroborated the results obtained by OCT imaging. NV could be detected infiltrating the sub-RPE space and the ONL. The photoreceptor inner segments attached to the ONL appear to be dragged upwards by the invading vessel (yellow arrow), also RPE migration along the invading vessel was observed (red arrows). The detachment of the photoreceptor layer from the RPE was most likely produced by the histological procedure. GCL: ganglion cell layer, IPL: inner plexiform layer, INL: inner nuclear layer, OPL: outer plexiform layer, ONL: outer nuclear layer, OLM: outer limiting membrane, I/OS: photoreceptor inner/outer segment border, RPE: retinal pigment epithelium, CH: choroid. Scale bar, 50 µm.

In two out of five eyes we could also detect the final stage, the “effector phase”, with induction of neovascularization (NV) (Fig. 10). All the alterations of retinal layer morphology were evenly distributed across the entire retina, no preferred localization could be observed. Accordingly, the sites of NV were detected in the central retina close to the optical disc (Fig. 10A) as well as in the periphery (Fig. 10D). Repeated *in vivo* examinations revealed no further sites of NV until 5 months of age and also the appearance of the NV did not change over time (data not shown). OCT analyses detected extensive alterations of retinal layer morphology (Fig. 10B,C,E) that were directly associated to the rupture of the RPE (Fig. 10B–F, arrow heads, bracket). Moreover, subretinal fluid accumulation was observed (Fig. 10E,F, asterisks). Neovascular processes are characterized by fenestrated vessels that can be visualized with *in vivo* SLO imaging^18^. Accordingly, by applying FL angiography we observed substantial extravasation of the dye from the fenestrated vessels at the sites of NV (Fig. 10A,D, arrows). Histological analysis of the respective NV by hematoxylin-staining (Fig. 10B, inset) corroborated the results obtained by OCT imaging (Fig. 10B,C). A vessel connected to the sub-RPE space was clearly detected along with hypertrophy of the surrounding RPE and RPE cells migrating along the invading vessel (Fig. 10B, inset, red arrows).

## Discussion

Norrie disease is caused by mutations affecting the *NDP* gene^1^. The typical clinical signs in the eye are bilateral retinal degeneration and extensive vitreous membranes^2^. Mutations in the *NDP* gene also account for a variety of other familial and sporadic diseases, including exudative vitreoretinopathy^33^, advanced retinopathy of prematurity^34^, and Coats disease^35^. A common feature of all these disorders is hypovascularization of the retina. These particular defects were correlated to the Wnt signalling pathway^36^ with Norrin interacting during development with Frizzled4, Lrp5^37^ and Tspan12^38^ on the surface of endothelial cells. Moreover, Wnt signalling is not only essential for correct vascular development but also for vascular homeostasis in the mature retina. Recently, it could be demonstrated that activation of the Wnt pathway induces retinal inflammation and oxidative stress^39^. Our results reported in the present study support these observations. For instance, in the mature retina of Ndph-knockout mice we also observed widespread inflammatory processes and sites of neovascularization that are hallmarks of various ocular diseases.

As we have shown recently, the initial vascular abnormalities due to Norrin deficiency developed to further profound vascular defects affecting vascular integrity and retinal function. Vascular remodelling was still ongoing until 2 months of age and was considered as an attempt to compensate for the lack of inner retinal capillaries^9^. Albeit no further vascular alterations of the superficial plexus in the adult and ageing retina were evident (Fig. 1), we observed profound changes of the retinal layer morphology (Figs 4, 5, 7 and 8) along with severe inflammatory processes (Figs 3, 7 and 8).

**Figure 11:**
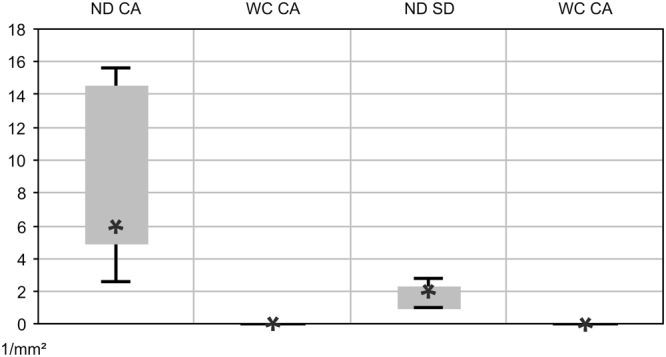
Quantification of retinal alterations observed in OCT imaging. Cystic alterations (CA) and subretinal defects (SD) were estimated per mm^2^ in the retina of 2-months-old Ndph^y/−^ mice (ND, n = 5) and of age-matched wild type controls (WC, n = 5). Data represent the mean ± SD.

*In vivo* OCT imaging detected various fluid-filled microcysts in the INL (Figs 4, 5 and 11) that, according to location, size and shape, closely resemble the cystic alterations in CME that occurs in various human retinal diseases^10,12–14^. In CME spherical or ovoid cysts are predominantly located in the INL of the perifoveal region^10,16^. Although the pathophysiology of CME is not yet completely understood, several pathological conditions like tissue hypoxia, inflammatory processes and a dysfunctional BRB have been associated to CME^10,11,16,23^. Moreover, Müller cells have been identified as a key player in cyst formation^10,16^. In the retina, the high metabolic activity of neuronal cells results in excessive water production. The water is then cleared via uptake by Müller cells and released into the blood or into the vitreous body^17,19,40^. Under pathological conditions swelling of Müller cells occurs which is believed to be induced by an altered expression of water channels. Particularly, it has been shown, that hypoxia directly influences the water channel expression pattern and thus the function of Müller cells in retinal water homeostasis^10,16^.

As we have demonstrated previously, the lack of inner retinal capillaries in the adult Norrin deficient retina resulted in long-term tissue hypoxia^9^. Here, we could now demonstrate that the observed microcystic alterations in the retina of adult Ndph^y/−^ mice were closely surrounded by activated Müller cells (Fig. 5). Taken together, it can be assumed that the hypoxic conditions in the Norrin depleted retina have severely altered Müller cell function that resulted in water accumulation and eventually cellular swelling comparable to the situation in human CME. Moreover, in the healthy retina Müller cells can usually not be visualized in OCT imaging. It seems, however, that the physiological alterations that have induced the microcystic lesions also enabled *in vivo* visualization (Fig. 5). Directly within or very close to the cystic lesions black roundish dots could be observed (Fig. 5A, magnifications 1–3). Since in the Norrin deficient retina no other structural entities could be present at these sites in this number we assume that these dots may represent the cell bodies of the Müller cells that are located within the INL (Fig. 9).

In addition, we here present evidence that even in a developmental disorder like Norrie disease, inflammation plays a crucial role for disease progression. Not only highly reactive Müller cell gliosis was observed (Fig. 3) but also the retinal microglial cells were affected. Under normal conditions microglial cells settle into the plexiform layers of the retina and gain a highly branched morphology with small cell bodies and long cellular protrusions (Fig. 7D). Once activated retinal microglia start to migrate and transform into large amoeboid cells^41^. In the adult Norrin deficient retina we could clearly observe enhanced proliferation and migration of activated microglial cells (Fig. 7C,E). Moreover, we suggested that also the microglial cells could be visualized in OCT *in vivo* imaging due to the activation-dependent morphological alterations of the cells (Fig. 7A,B). As it is known from mouse models for retinal degeneration, activated microglial cells primarily accumulate and form clusters at sites of ongoing disease progression^41^. Here, in the Norrin deficient retina progressive pathological alterations were particularly observed within the INL. Beside the microcystic lesions (Figs 4 and 5) and the associated activated Müller cells (Fig. 5) OCT imaging revealed large structures spanning the entire INL (Fig. 7A,B). On the basis of the facts described above we assume that we were able to *in vivo* visualize clusters of activated microglial cells by OCT imaging.

**Figure 12:**
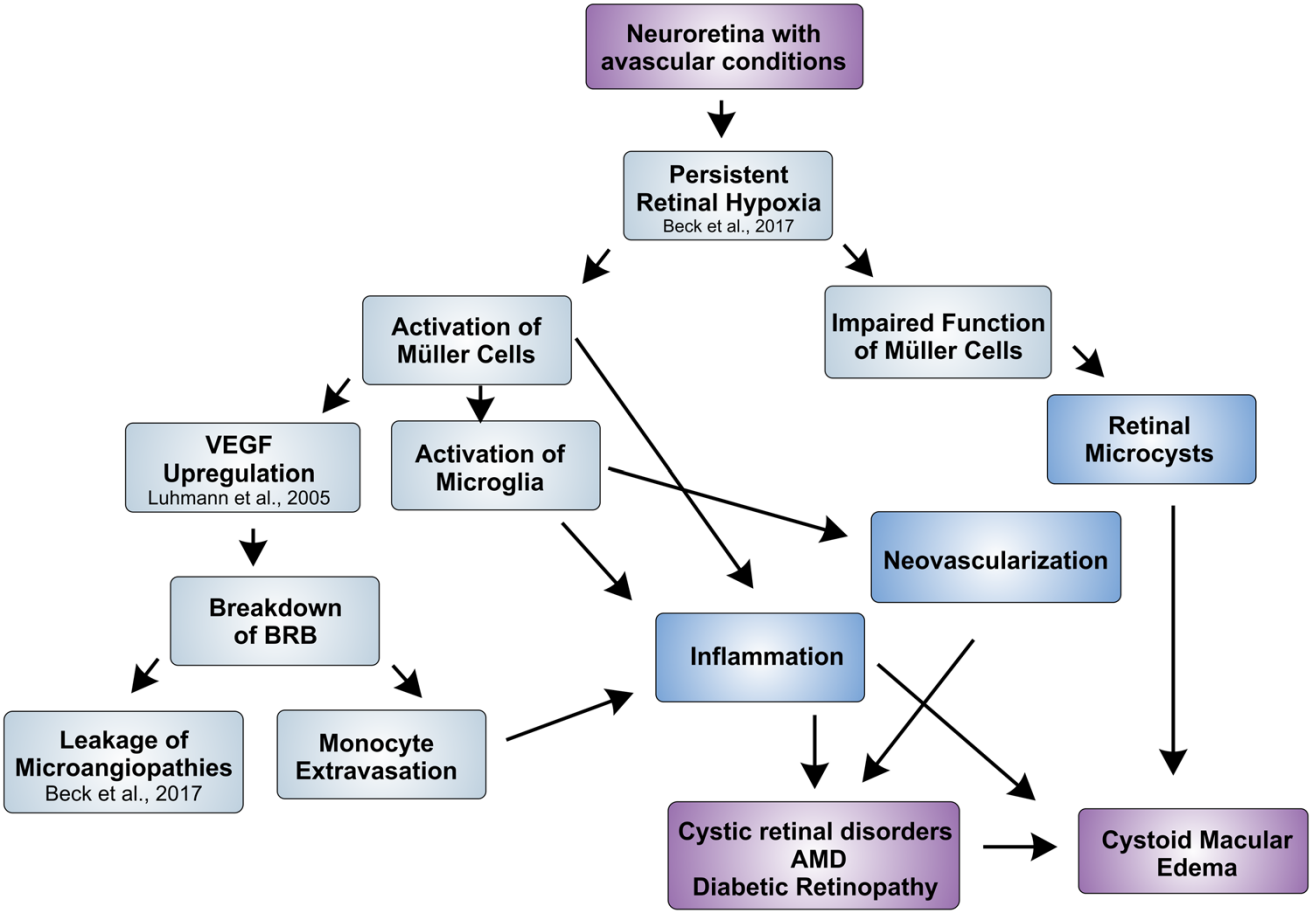
Schematic overview on the pathology of the aged Norrin deficient retina. Very similar to the human macula, in Ndph^y/−^ mice the fully developed neuroretina with its high metabolic demands meets an avascular environment due to the lack of inner retinal capillaries and therefore develops hallmarks of AMD and cystoid macular edema.

Interestingly, we also observed several subretinal defects which we suggest to correspond to activated microglia in the outer retina (Figs 8 and 11). Actually, a common hallmark of pathophysiological alterations in retinal disorders are microglial cells that infiltrate the outer retina, displace the photoreceptor cells, rupture the RPE and induce a local inflammatory response which finally results in breakdown of the Bruch’s membrane and choroidal neovascularization^26^. Very likely, similar event-associated pathogenic processes may have triggered neovascularization in aged Ndph^y/−^ mice. In OCT imaging, blood vessels were clearly visible disrupting the RPE and the photoreceptor layer (Fig. 10). Similar inflammatory processes that involve activated microglia cells play a central role in neovascularization in the development of wet AMD^42^. Wet AMD is less common than dry but is the most severe form and accounts for the majority of vision loss^43^. The unique anatomy of the human macula accounts for its particular vulnerability. The avascular neuroretina of the macula has to meet the great metabolic demands of an extremely high cell count. Mice do not have a specialized retinal region like the macula^44^, however, retinal Norrin deficiency create a very similar situation: the high metabolic demand of a fully developed neuroretina meets the complete lack of inner retinal capillary layers which, in the long-term, have resulted in the observed retinal alterations. Moreover, the observed microcysts in the retina of aged Ndph^y/−^ mice reflect macular edema which is associated to many ocular diseases like AMD, diabetic retinopathy and retinal vein occlusion^10^. Taken together, the Norrin deficient retina of aged Ndph^y/−^ mice reflects not only single aspects of ocular disorders but several of the complex processes in pathophysiological alterations including metabolic homeostasis, neovascularization and inflammation (Fig. 12). Thus, aged Ndph^y/−^ mice could be a suitable small animal model not only for research but also for therapy development in cystic retinal disorders.

Moreover our results suggest inflammation as a common process that plays a key role in many if not all ocular disorders. Due to the blood retina barrier which consists of both an inner and an outer barrier the eye has traditionally been considered as immune privileged. However, during the last decades there is growing evidence that inflammation plays an important role in ocular diseases, even in disorders that develop from completely different pathophysiological processes, amongst these are the inherited retinal degenerative disease retinitis pigmentosa^45^, the metabolic disorder diabetic retinopathy^46^ and the age related condition AMD^47^. In the present work we now present evidence that inflammation also plays a crucial role in a developmental vascular disorder, namely Norrie disease. We therefore propose that inflammation can be considered as a common process that develops independent from the initial cause of the disease. Furthermore, on the basis of the results obtained in the aged Norrin deficient retina we assume that an inflammatory response is evoked over time in the chronic phase of the disease. This however opens up new options for treatment. On the one hand, existing therapies might benefit from a supplementary treatment directed to the immune response to alter the severity and course of the disease. On the other hand, anti-inflammatory therapies might be considered also in disorders involving impaired vascular homeostasis and neovascularization either acquired like in diabetic retinopathy or inherited like in Norrie disease.

## Methods

### Ethics statement

All procedures were performed in accordance with the local ethics committee (Regierungspraesidium Tuebingen), German laws governing the use of experimental animals, and the ARVO statement for the use of animals in ophthalmic and visual research. The Institute of Animal Welfare and the Veterinary Office at the University of Tuebingen ensures compliance with all applicable regulations for the use of animals. All examinations are approved by The Institute of Animal Welfare and the Veterinary Office at the University of Tuebingen and the Regierungspraesidium Tuebingen.

### Animals

The *Ndp*^*y*/−^ mouse line was generated by Berger *et al*. as described previously^3^. The mutation is kept on a C57BL/6 J background. Genotyping was performed by PCR analysis of ear DNA^3^. Animals were kept under a 12 hours light/dark cycle and had free access to food and water.

### *In vivo* analysis of retinal layer structure

OCT and cSLO were performed consecutively in the same session. Mice were anesthetized with ketamine (66.7 mg/kg) and xylazine (11.7 mg/kg) and pupils dilated with tropicamide (Mydriaticum Stulln, Pharma Stulln, Stulln, Germany). OCT analyses were performed with a commercially available Spectralis™ HRA + OCT device (Heidelberg Engineering, Heidelberg, Germany) featuring a broadband superluminescent diode low coherent light source^24,25^. Each two-dimensional B-Scan (set to 30° field of view) consisted of 1536 A-scans acquired at 40,000 scans/second. Imaging was performed using the proprietary software package Eye Explorer (version 5.3.3.0., Heidelberg Engineering) Images were processed and arranged with Corel Draw X3 (Ottawa, Canada). For detailed high-resolution imaging of the mouse retina we used the black on white mode which presents high reflectivity as dark shade and low reflectivity with light shade as we have published previously^24,25^.

### *In vivo* angiography

Scanning-Laser Ophthalmoscopy (SLO) was performed after Optical Coherence Tomography (OCT) examination. Retinal structures of the anesthetized animals were visualized with an HRA 2 (Heidelberg Engineering, Heidelberg, Germany) according to a previously published method^18^. Briefly, the HRA 2 system features lasers in the short (visible) wavelength range (488 nm and 514 nm), and also in the long (infrared) wavelength range (785 nm and 815 nm). To monitor the vascular changes in the eyes of 2 months old *Ndph*^*y*/−^ mice *in vivo*, we used fluorescein (FL) as a dye (Fluorescein 10%, Alcon) with the argon blue laser (488 nm; barrier, 500 nm) and indocyanine green (ICG, Pulsion medical system AG) with the infrared laser (795 nm; barrier 800 nm).

### Analyses of retinal whole mount preparations

For collection of the eyes the mice were euthanised by CO_2_ inhalation. For whole mount preparations eyes were fixed in 4% PFA for 2 h, and retinas were dissected and washed three times with PBS for 1 hour, then incubated in permeabilisation buffer (1% BSA, 0.5% Triton-100 in PBS, for double staining with 5% serum, from which the second antibody was made) for 1 hour at room temperature. Retinas were incubated in Lectin-FITC or rabbit anti-glial fibrillary acidic protein antibody (GFAP; polyclonal GFAP antibody; rabbit anti-mouse 1 mg/mL; Dako, Hamburg, Germany) and tetramethylrhodamine isothiocynate (TRITC)-labeled isolectin B4 (Sigma-Aldrich, München, Germany; dilution 1:50) over night at 4 °C. A FITC-labeled anti-rabbit-antibody (1:20, Dako) was used for the detection of GFAP primary antibody. After washing three times for 1 hour with PBS, the samples were covered. Photographs were taken with a microscope connected to a video camera (Leica, Wetzlar, Germany).

### Retinal digest preparations

Retinal vascular preparations were performed using a trypsin digestion technique as previously described^48^. Briefly, the retinas were fixed in 4% formalin for 24 h and subsequently incubated in 3% trypsin solution resolved in 0.2 mol/L Tris buffer (pH 7.4) for 120 min. For analysis of intraretinal vasculature, the vessels above the inner limiting membrane were carefully removed. Subsequently, the retinal digest preparations were carefully washed with aqua bidest and flat mounted on slides. Finally, the samples were stained using periodic-acid Schiff reagent (PAS). The vasculature was examined using retinal image analysis (Analysis Pro System; Olympus Opticals, Hamburg, Germany). For analysis of accumulation of extravascular cells of the mononuclear phagocyte system the retinas were first subjected to whole mount staining with Lectin-FITC and photographed. Subsequently, after fixation in 4% formalin for 24 h, the retinas were subjected to retinal digestion and PAS staining and photographs of the corresponding areas were taken.

### Immunohistochemistry

Immunohistochemical analyses were performed as described previously^27,49^. Briefly, 10 μm retinal sections were incubated with primary antibodies at 4 °C. Antibodies included rabbit anti-Iba1 antibody (Wako Chemicals, Neuss, Germany), rabbit anti-TSPO antibody (Abcam, Cambridge, UK), and goat anti-GFAP antibody (Santa Cruz Biotechnology). After washing, samples were labelled with a secondary antibody conjugated to Alexa488 (green) or Alexa594 (red) (Jackson Immuno-Research, West Grove, PA, USA) and counterstained with DAPI. A Lectin-TRITC conjugate (Merck, Darmstadt, Germany) was used for vessel detection. Sections were mounted in DAKO fluorescent mounting medium (Dako Deutschland GmbH, Hamburg, Germany) and viewed with an Axioskop2 MOT Plus Apotome microscope (Carl Zeiss).

### Histology

The eye was fixed in 4% paraformaldehyde and the eyeucps isolated. After washing in PBS, the eyecups were incubated overnight at 4 °C in 30% sucrose and afterwards cryoprotected in OCT compound. The morphology of the retina, RPE and choroid was visualized in hematoxylin-stained cryosections.
